# Development of MOF-derived Co_3_O_4_ microspheres composed of fiber stacks for simultaneous electrochemical detection of Pb^2+^ and Cu^2+^

**DOI:** 10.1007/s00604-024-06623-7

**Published:** 2024-08-17

**Authors:** Jieli Guo, Jin Li, Xiujing Xing, Wei Xiong, Hao Li

**Affiliations:** 1 Key Laboratory of Novel Biomass-Based Environmental and Energy Materials in Petroleum and Chemical Industry, Hubei Key Laboratory of Novel Reactor & Green Chemical Technology, School of Chemistry and Environmental Engineering, Wuhan Institute of Technology, Wuhan, 430205 China; 2 Chemistry Department, University of California, Davis, 95616 USA; 3 Advanced Institute for Materials Research (WPI-AIMR), Tohoku University, Sendai, 980-8577 Japan

**Keywords:** Transition metal oxides, Metal-organic frameworks, Heavy metal ions, Electrochemical sensors, Modified glassy carbon electrode, Differential pulse anodic stripping voltammetry

## Abstract

As an ideal transition metal oxide, Co_3_O_4_ is a P-type semiconductor with excellent electrical conductivity, non-toxicity and low cost. This work reports the successful construction of Co_3_O_4_ materials derived from metal-organic frameworks (MOFs) using a surfactant micelle template-solvothermal method. The modified electrodes are investigated for their ability to electrochemically detect Pb^2+^ and Cu^2+^ in aqueous environments. By adjusting the mass ratios of alkaline modifiers, the morphological microstructures of Co_3_O_4_-X exhibit a transition from distinctive microspheres composed of fiber stacks to rods. The results indicate that Co_3_O_4_-1(NH_4_F/CO(NH_2_)_2_ = 1:0) has a distinctive microsphere structure composed of stacked fibers, unlike the other two materials. Co_3_O_4_-1/GCE is used as the active material of the modified electrode, it shows the largest peak response currents to Pb^2+^ and Cu^2+^, and efficiently detects Pb^2+^ and Cu^2+^ in the aqueous environment individually and simultaneously. The linear response range of Co_3_O_4_-1/GCE for the simultaneous detection of Pb^2+^ and Cu^2+^ is 0.5–1.5 μM, with the limits of detection (LOD, S/N = 3) are 9.77 nM and 14.97 nM, respectively. The material exhibits a favorable electrochemical response, via a distinctive Co_3_O_4_-1 microsphere structure composed of stacked fibers. This structure enhances the number of active adsorption sites on the material, thereby facilitating the adsorption of heavy metal ions (HMIs). The presence of oxygen vacancies (O_V_) can also facilitate the adsorption of ions. The Co_3_O_4_-1/GCE electrode also exhibits excellent anti-interference ability, stability, and repeatability. This is of great practical significance for detecting Pb^2+^ and Cu^2+^ in real water samples and provides a new approach for developing high-performance metal oxide electrochemical sensors derived from MOFs.

**Graphical abstract:**

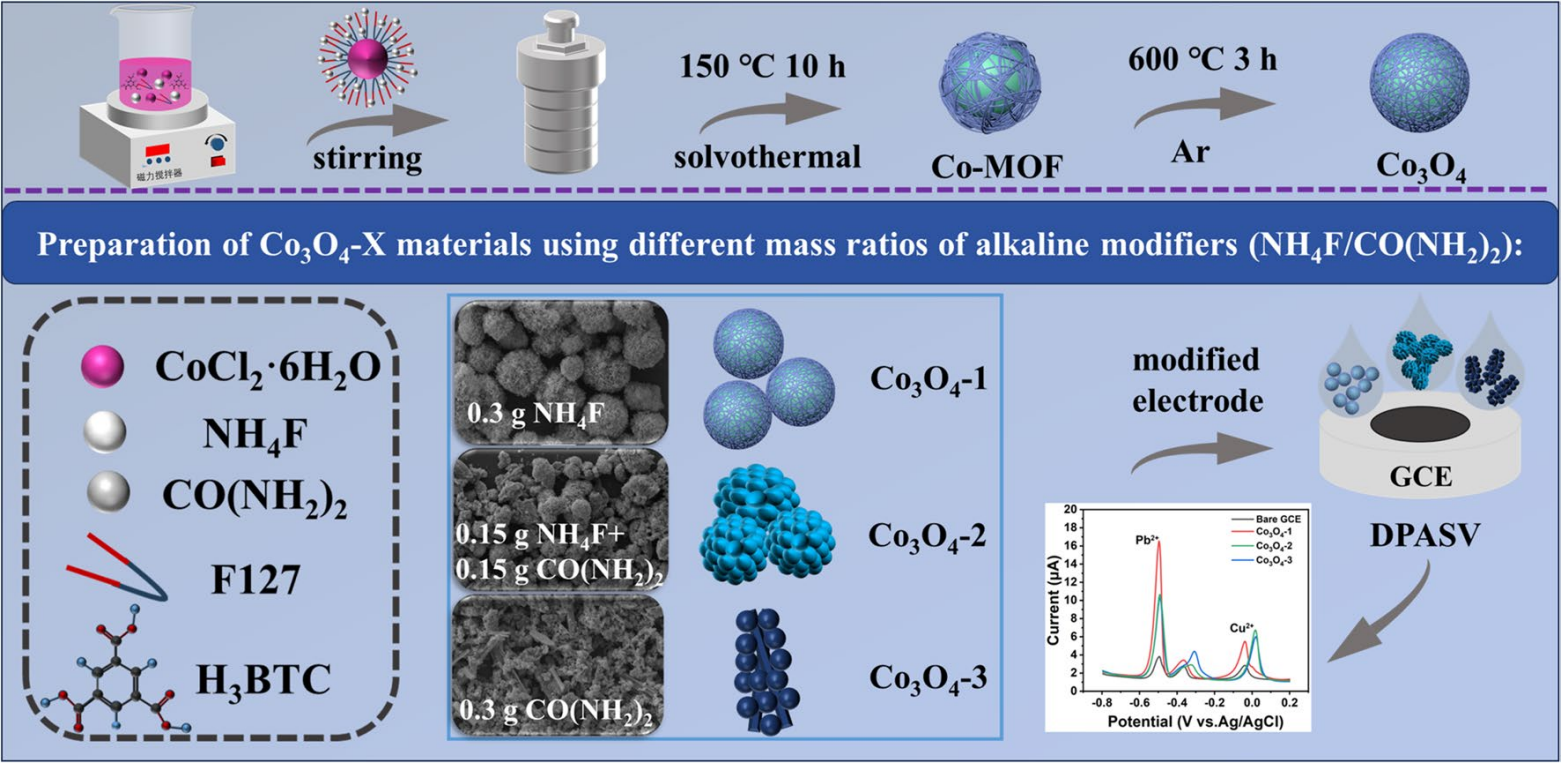

**Supplementary information:**

The online version contains supplementary material available at 10.1007/s00604-024-06623-7.

## Introduction

In recent years, the discharge of heavy metal ions (HMIs) from industrial effluents has increased due to rapid economic development. Excessive HMIs can pose a serious threat to ecosystems and human health [1–3]. Therefore, it is crucial to detect HMIs and determine their levels in natural and drinking water bodies [4, 5]. A variety of methods have been reported for the detection of HMIs [6, 7], such as atomic fluorescence spectrometry, atomic absorption spectrometry, atomic emission spectrometry, ultraviolet-visible spectrophotometry, inductively coupled plasma mass spectrometry, and X-ray fluorescence spectrometry. Although these detection methods have been widely used, their use is limited due to the complexity and high cost of operation procedures [8]. In contrast, electrochemical analytical methods have the advantages of simple operation, low cost, fast analytical speed, high sensitivity, strong anti-interference, and real-time on-line detection of HMIs in the aqueous environment [9], and are widely used in the field of electrochemical detection of HMIs [10–12]. Therefore, there is a requirement for the research and development of low-cost, sensitive, and practical materials to detect HMIs in aqueous environments [13–15].

Metal-organic framework (MOF)-derived metal oxides are well-known for their tunable structure, high porosity, high electrochemical activity, and excellent stability. These properties make them promising materials for modified electrodes and have garnered significant attention in the field of electrochemical sensors [16, 17]. Specifically, MOF-derived metal oxide semiconductors, such as Co_3_O_4_ [18], NiO [19], CuO [20] and FeO [21], are low-toxicity, inexpensive, and have a large specific surface area and rich pore structure [22]. Among them, Co_3_O_4_ is a mixed-valence transition metal oxide with a spinel structure that exhibits distinctive P-type semiconductor properties. It is composed of Co^2+^ and Co^3+^ ions occupying tetrahedral and octahedral positions, respectively. They are commonly used in catalysis [23], lithium-ion batteries [24], and electrochemical sensors [25] due to their redox activity and Co^2+^/Co^3+^ ratio in cationic sites. Currently, many studies have been conducted to improve the properties of materials by modulating the morphology of MOF-derived Co_3_O_4_. For example, Lei et al. [26] reported the successful synthesis of a series of mesoporous Co_3_O_4_ catalysts for toluene oxidation by pyrolysis of different forms of Co-MOFs. The catalyst displayed varying physicochemical properties, including exposure surface, Co^2+^/Co^3+^ specific surface area, and low-temperature reducibility. In catalytic tests, the Co_3_O_4_ catalysts demonstrated varying degrees of catalytic activity and stability in the oxidation of toluene to carbon dioxide and water. Shao et al. [27] reported Co_3_O_4_ and graphene foam (GF) derived from a MOF with various morphologies. The Co_3_O_4_/GF material can serve as a direct anode for lithium-ion batteries, exhibiting superior electrochemical performance compared to pure Co_3_O_4_ in terms of specific capacity, long cycle stability, and multiplicity performance. In addition, Yang et al. [28] reported the preparation of MOF-derived leafy and rod-shaped Co_3_O_4_ for electrochemical sensing of H_2_O_2_ using leaf-like zeolitic imidazolate framework and stick-like zeolitic imidazolate framework. Leafy Co_3_O_4_ showed good electrocatalytic activity for H_2_O_2_ in the ranges of 0.5–4500 and 4500–10000 μM, with a detection limit of 0.47 μM. rod Co_3_O_4_ showed good H_2_O_2_ detection performance in the ranges of 0.5–4500 and 4500–10000 μM, with a detection limit of 0.33 μM. The constructed sensors also showed excellent anti-interference ability, repeatability, stability and applicability. However, there have been fewer studies aimed at improving the performance of electrochemically detected HMIs by modulating the morphology of Co_3_O_4_ through MOF growth mechanisms. These mechanisms include the use of surfactants [29], coordination modes of metal ions and organic linkers [30], and alkaline modifiers [31], among others. Therefore, this work focuses on the effect of morphology modulation of MOF-derived Co_3_O_4_ materials on the performance of detecting Pb^2+^ and Cu^2+^ in aqueous environments.

This study utilized the surfactant micelle template-solvent thermal method to construct MOF-derived Co_3_O_4_ microspheres. The detection performance of HMIs in the field of electrochemical sensors was analyzed by modulating the morphology and electronic structure of Co_3_O_4_ through varying different mass ratios of alkaline modifiers.

The modified electrode utilized Co_3_O_4_-1/GCE as the active material and exhibited a linear response range of 0.5–1.5 μM for the simultaneous detection of Pb^2+^ and Cu^2+^. The limits of detection (LOD, S/N = 3) were 9.77 nM and 14.97 nM, respectively. The material exhibits an excellent electrochemical response that can be attributed to its distinctive microsphere structure composed of stacked fibers. Due to the oxygen vacancy (O_V_) generated by the change of cobalt ions’ mixed valence state, this provides new ideas for realizing high-performance electrochemical sensors for MOF-derived metal oxides.

## Experimental section

### Chemicals and materials

Cobalt chloride hexahydrate (CoCl_2_·6H_2_O), urea (CO(NH_2_)_2_), potassium chloride (KCl), anhydrous ethanol (C_2_H_5_OH), anhydrous sodium acetate (NaAc), acetic acid (HAc), N,N-dimethylformamide (DMF), Potassium ferricyanide (K_3_[Fe(CN)_6_]) and potassium ferricyanide (K_4_Fe(CN)_6_·3H_2_O) were purchased from Sinopharm Chemical Reagent Co. Homotricarboxylic acid (H_3_BTC) and 5 wt% nafion solution were purchased from Shanghai Aladdin Biochemical Technology Co. Ammonium fluoride (NH_4_F), polyether F127 (F127), sodium dihydrogen phosphate dihydrate (NaH_2_PO_4_·2H_2_O) and disodium hydrogen phosphate dodecahydrate-dodecahydrate (Na_2_HPO_4_·12H_2_O) were purchased from Shanghai McLean Biochemistry Technology Co. Lead standard solution (1000 μg/mL) and copper standard solution (1000 μg/mL) were purchased from the National Center for Analysis and Testing of Nonferrous Metals and Electronic Materials. 0.05 μm alumina powder was purchased from Zhejiang Lixie Instrument Co. Ultrapure water was used for all aqueous solutions. All chemical reagents were of analytical grade and did not require further purification.

### Preparation of Co-MOF precursors

**Scheme 1:**
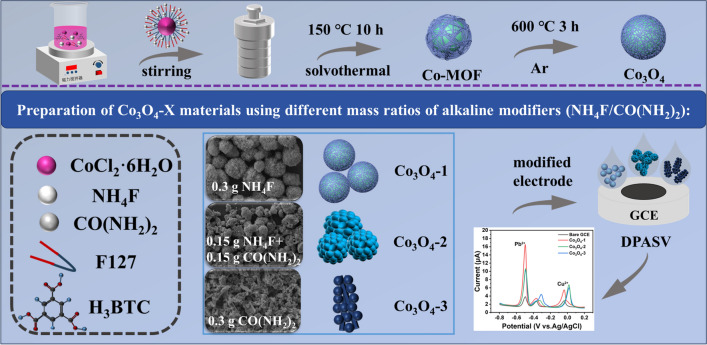
Schematic representation of the formation of Co_3_O_4_-X

Synthesis of MOF-derived Co_3_O_4_ materials (Scheme 1) was achieved via a surfactant micelle template-solvothermal method [32]. Based on the methodology reported in the literature, we made certain modifications to it. First, 1 mmol of CoCl_2_·6H_2_O, 1.5 mmol of H_3_BTC, 0.5 g of F127 and 0.3 g of NH_4_F (or 0.15 g of NH_4_F and 0.15 g of CO(NH_2_)_2_ and 0.3 g of CO(NH_2_)_2_) were ultrasonically dissolved in 50 ml of DMF-water solution (v/v = 1:1), and a homogeneous magenta color solution could be obtained by stirring at room temperature for 30 min. The above solution was then transferred to a 100 ml Teflon-lined autoclave and kept at 150 ℃ for 10 h. After natural cooling to room temperature, the product was collected by centrifugation, the precipitate was washed three times alternately with ethanol and water. And the product was dried at 60 ℃ for 12 h, resulting in a magenta-colored Co-MOF precursor. Different mass ratios of alkaline conditioner (NH_4_F/CO(NH_2_)_2_) were made on the base of the above, different mass ratios as X = 1:0, 1:1, 0:1, respectively.

### Preparation of Co_3_O_4_ materials

Each of the above powders was placed in a porcelain boat. The porcelain boat was then placed in a tube furnace for heat treatment at a temperature of 600 ℃ (V _rise_ = 2 ℃/min, Ar), kept for 3 h, and subsequently reduced to room temperature at a rate of 5 ℃/min. The Co_3_O_4_-X material (X = 1:0, 1:1, 0:1) were obtained. The materials were labelled as Co_3_O_4_-1, Co_3_O_4_-2, and Co_3_O_4_-3, based on their mass ratios of NH_4_F and CO(NH_2_)_2_ of 1:0, 1:1, and 0:1, respectively. The Co_3_O_4_ material without F127 (NH_4_F/CO(NH_2_)_2_ = 1:0) was prepared using the identical synthetic pathway and labelled as Co_3_O_4_-4.

### Characterization instruments and electrochemical testing

The morphology of the materials was observed using a scanning electron microscope (SEM). The microcrystalline structure and composition of the materials were determined using an X-ray diffractometer (XRD) with Cu-targeted Kα radiation. The chemical compositions and valence changes of the surface of the samples were analyzed using an X-ray photoelectron spectrometer (XPS).

The electrochemical tests and characterizations in this experiment were conducted using a CHI850D electrochemical analyzer from Shanghai Chenhua Instrument Co. Electrochemical characterization and testing were performed using a three-electrode system. The working electrode was either a bare glassy carbon electrode (GCE) or a modified electrode. The reference electrode was a saturated Ag/AgCl electrode and the counter electrode was a platinum wire electrode, all of which were purchased from Shanghai Chenhua Instrument Co. The three electrodes were assembled as a system for cyclic voltammetry (CV), electrochemical alternating impedance spectroscopy (EIS), differential pulsed anodic dissolution voltammetry (DPASV), and chronocoulometric coulometry (CC). The electrode materials were electrochemically characterized in 5 mM [Fe(CN)_6_]^3−/4−^ with 0.1 M KCl, and electrochemical tests were conducted in a 0.1 M pH = 5.0 NaAc-HAc buffer.

### Preparation of working electrode

Before modifying the electrodes, the bare GCE were polished with 0.05 μm alumina powder, rinsed several times with deionized water and dried them with a wash ball. To prepare the working electrode, 2 mg of the sample were dispersed in 500 μl of deionized water, 490 μl of anhydrous ethanol, and 10 μl 5 wt% of Nafion solution and sonicated the mixture for 1 h to form a well-dispersed suspension. 4 μl of the suspension was added dropwise to the surface of the bare GCE. The electrode was then dried at room temperature to obtain a modified Co_3_O_4_-X/GCE electrode.

## Results and discussion

### Morphological and structural characterization

**Fig. 1:**
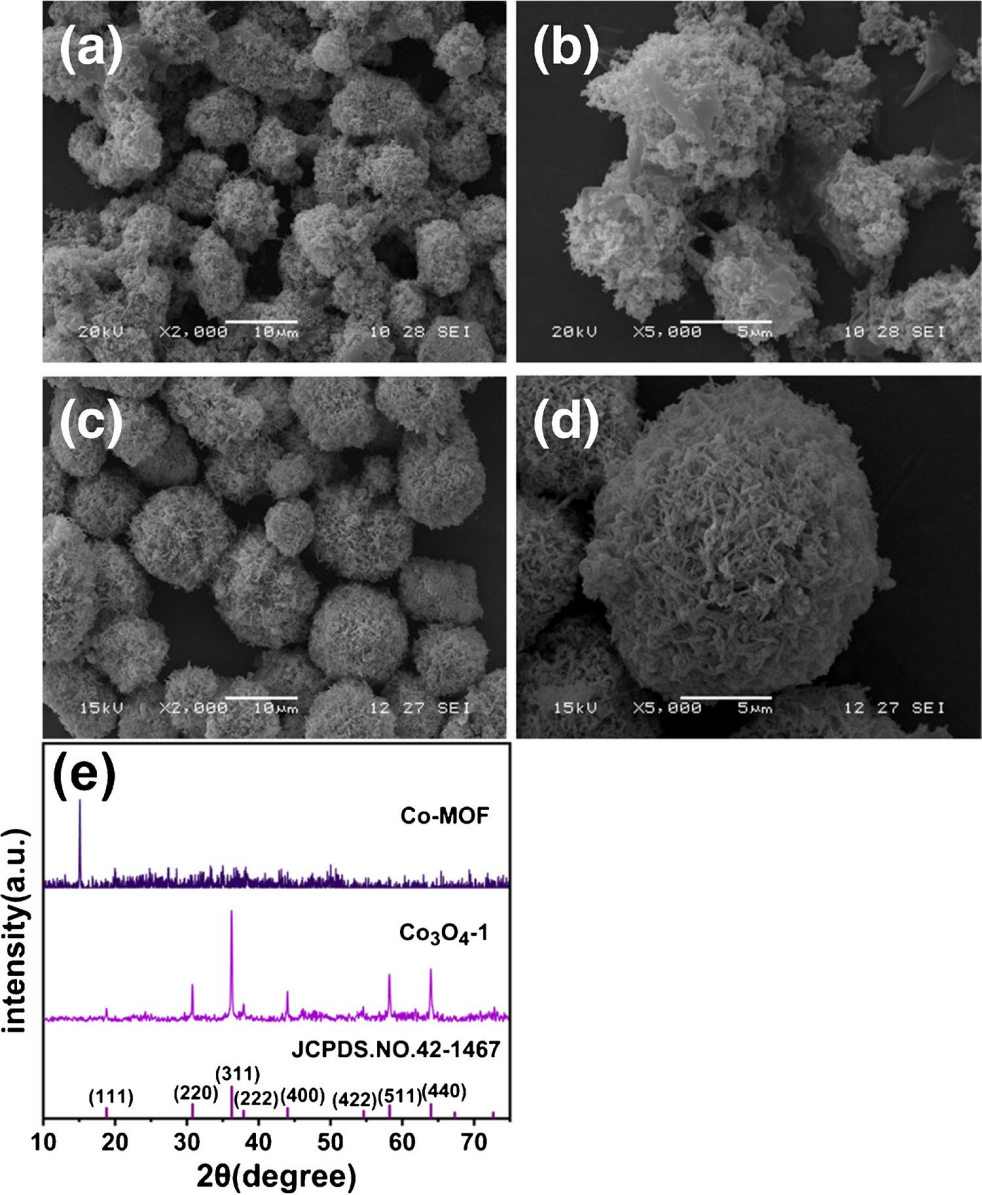
(**a-b**) SEM images of Co-MOF, (**c-d**) SEM images of Co_3_O_4_-1, (**e**) XRD plots of Co-MOF and Co_3_O_4_-1

SEM characterization is used to analyze the microscopic morphology of the samples before and after heat treatment. Before heat treatment, the Co-MOF precursor is in the form of outwardly dispersed microspheres, as shown in Fig. 1a-b. The surface of the microspheres is made up of fibers piled up in a more irregular shape. After the high-temperature heat treatment, Co_3_O_4_-1 exhibits a distinctive microsphere structure composed of stacked fibers with a uniformly dispersed shape, similar to the morphology of the Co-MOF precursor (Fig. 1c-d). Co_3_O_4_-1 maintains the original skeletal structure of the Co-MOF precursor. The particle size distributions of Co-MOF and Co_3_O_4_-1 are presented in Fig. S2a-b. The mean diameter of Co-MOF is approximately 9–10 μm, while that of Co_3_O_4_-1 is around 12–13 μm. The particle size of Co_3_O_4_-1 becomes larger (Fig. S2b). This may be attributed to the collision between Co-MOF particles during high temperature heat treatment with the increase of temperature and spontaneous aggregation to reduce the surface Gibbs free energy. This leads to the formation of microspheres with larger particles [33]and a stable aggregation state. This statement is in line with the literature by Shen’s group [34] in which they report that using MOF as a precursor and synthesizing metal oxides through heat treatment can preserve the original skeletal structure of the MOF precursor.

To investigate the crystal structure and elemental composition of Co-MOF and Co_3_O_4_-1, samples were subjected to XRD characterization prior to and following heat treatment. As shown in Fig. 1e, the Co-MOF precursor before heat treatment exhibits an intense and sharp diffraction peak at 2θ = 15.0° resulting from the coordination of H_3_BTC and transition metal cations. This is consistent with the simulated profiles of Co-MOF in the literature [35], indicating that they have similar crystal structures. The sharp and intense diffraction peaks of Co_3_O_4_-1 near 19.0°, 31.3°, 36.8°, 38.5°, 44.8°, 55.6°, 59.4°, and 65.2° after heat treatment are clearly observed in Fig. 1e corresponds to the (111), (220), (311), (222), (400), (422), (511) and (440) [36] crystal planes of the standardized card for the structure of Co_3_O_4_ spinel (JCPDS. No. 42–1467). No additional diffraction peaks are observed, thereby indicating the successful synthesis of Co_3_O_4_.

**Fig. 2:**
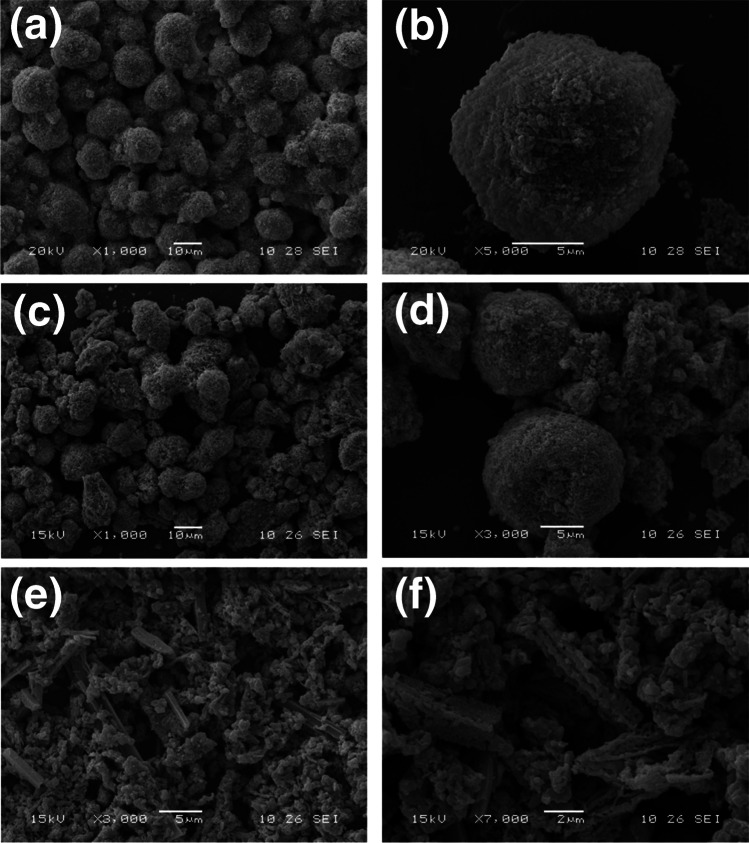
(**a-b**) SEM images of Co_3_O_4_-4 without surfactant F127, (**c-d**) SEM images of Co_3_O_4_-2, (**e–f**) SEM images of Co_3_O_4_-3

To investigate the formation mechanism of Co_3_O_4_-1 microspheres, it might be related to the addition of surfactant F127. As shown in Fig. 2a-b, in the absence of surfactant F127, Co_3_O_4_-4 exists in the form of microspheres comprising small particles arranged in a relatively homogeneous manner, although there is a certain degree of agglomeration [37]. In contrast, Co_3_O_4_-1 with the addition of surfactant F127 exhibits a distinctive microsphere structure and the surface of the microspheres is composed of stacked fibers with a uniformly dispersed shape (Fig. 1c-d). According to other literature [38], the amphiphilic surfactant F127 is not only able to form micelles of PPO cores surrounded by PEO shells in DMF-water (v/v = 1:1) solution, but also prevents agglomeration of Co-MOF crystals. In the absence of the surfactant F127, the carbonized Co-MOF is composed of Co_3_O_4_ particles and a cobalt metal-free carbon matrix during high-temperature thermal decomposition. This produces magnetic particles that act as catalysts, promoting of carbon rearrangement to form a special core-shell structure. Ultimately, inhomogeneous ordinary microspheres are formed and agglomeration occurs [39]. The addition of surfactant F127 results in the aggregation of hydrophilic chains on the exterior of the micelles due to the longer hydrophilic chains of the surfactant F127. Eventually, a distinctive microsphere structure comprising a fibrous build-up is formed, exhibiting a uniformly dispersed shape.

In addition, the alkaline modifiers may have some effect on the morphology. Therefore, in this work, CO(NH_2_)_2_ was selected as the alkaline modifier in place of NH_4_F, and the effect of varying mass ratios of alkaline modifiers on the morphology of Co_3_O_4_ was investigated [40]. As shown in Fig. 2c-f, the morphological microstructures of both Co_3_O_4_-2 and Co_3_O_4_-3 exhibit a range of morphologies, from distinctive microspheres to rods. When the mass ratio of NH_4_F/CO(NH_2_)_2_ was added as 1:1, it could be seen that a small part of the nanoparticles had an approximate spherical shape with an irregular morphology. When the mass ratio of NH_4_F/CO(NH_2_)_2_ was added as 0:1, the morphology and structure of the material changed significantly, with most of the shapes being granular and a small part of the shapes being rod-like. In comparison to the addition of NH_4_F/CO(NH_2_)_2_ in a mass ratio of 1:0 (Fig. 1c-d), the Co_3_O_4_-2 and Co_3_O_4_-3 materials exhibit a less homogeneous shape. Based on the above results, we speculate that the addition of NH_4_F plays a major role in the formation of Co_3_O_4_ microspheres. Due to the spatial coordination effect between cobalt ions and organic linkers, the fluoride ions in NH_4_F can also coordinate with the cobalt ions, and the fluoride ions are employed to intervene and reorganize the Co-MOF into a stable structure [41]. Therefore, the use of the alkaline modifier NH_4_F was able to regulate the growth kinetics of Co-MOF crystals, resulting in more uniform nucleation of Co-MOF on the micelles of the block polymer. Aggregation of hydrophilic chains on the outside of the micelles, leading to the formation of uniformly dispersed Co_3_O_4_ microspheres [42].

**Fig. 3:**
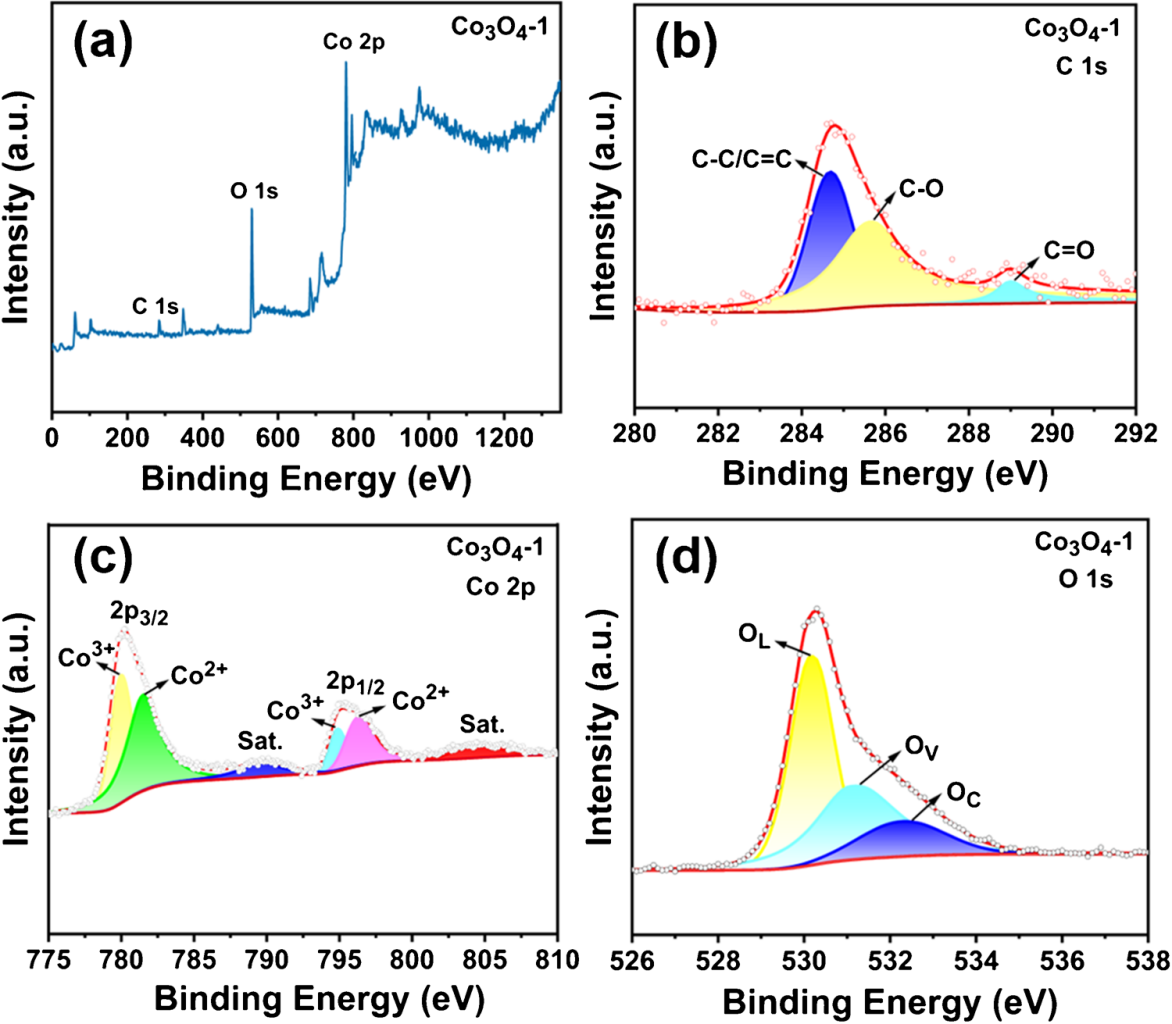
(**a**) XPS full spectrum, (**b**) C 1 s map, (**c**) Co 2p map and (**d**) O 1 s map of Co_3_O_4_ -1

To analyze the surface composition and elemental valence distribution of the samples, the Co_3_O_4_-1 sample is subjected to XPS characterization. Figure 3a presents the complete XPS spectrum of Co_3_O_4_-1, exhibiting clear signals from Co 2p, C 1 s, and O 1 s. These results demonstrate that Co_3_O_4_-1 is predominantly comprised of Co and O, with minimal contributions from other elements. This finding is consistent with the results of XRD characterization. As shown in Fig. 3b, the C 1 s map indicates that the binding energies are located at approximately 284.7 eV, 285.7 eV, and 289 eV. These energies correspond to three characteristic peaks matching C, which are C–C/C = C, C-O, and C = O bonds, respectively. These findings are consistent with those reported in reference [43].

The Co 2p spectrum of the Co_3_O_4_-1 sample is shown in Fig. 3c, exhibiting two principal peaks at approximately 780.4 eV and 795.5 eV, respectively. These peaks are attributed to the Co 2p_3/2_ and Co 2p_1/2_ spin states, which are the result of spin-orbit coupling in Co_3_O_4_. Furthermore, the two peaks are divided into two pairs of strong characteristic peaks, namely Co^3+^ and Co^2+^. The fitted peaks with binding energies located around 780.0 eV and 794.9 eV are attributed to Co^3+^, while the fitted peaks around 781.6 eV and 796.4 eV are attributed to Co^2+^. This suggests that Co has both divalent and trivalent oxidation states. As a result of high temperature calcination under inert atmosphere, part of Co^3+^ on the surface of Co_3_O_4_ is thermally reduced to Co^2+^ by carbon, leading to the generation of O_V_ on the surface of the material. This surface modification is conducive to the adsorption of HMIs during the electrochemical detection process. The Co^2+^/Co^3+^ ratio of this sample is estimated to be 1.26 by calculating the integrated area of the corresponding peak of Co_3_O_4_-1. This indicates that the material contains a greater proportion of Co^2+^. The appearance of two oscillating satellite peaks around 789.6 eV and 804.8 eV indicate that the structure of Co_3_O_4_ belongs to spinel [44, 45].

To further substantiate the presence of O_V_ [46], the O 1 s distribution of the Co_3_O_4_-1 sample is subjected to analysis. Figure 3d shows the primary peak centered at approximately 530.4 eV. The spectrum is fitted to three distinct peaks that corresponded to the following states in the oxide: lattice oxygen (O_L_), oxygen vacancy (O_V_) and chemisorbed oxygen (O_C_) [47]. The characteristic peaks, corresponding to 530.2 eV, 531.2 eV, and 532.4 eV, respectively, are evident in the figure. The results show that the oxygen on the surface of the samples mainly existed in the form of Co–O, O_V_, and O_2_. The O_V_/O 1 s peak area ratio of Co_3_O_4_-1 is calculated to be 0.42. Therefore, the XPS mapping analysis reveal that Co_3_O_4_-1 produce O_V_ following high-temperature heat treatment. This provides a favorable basis for the subsequent exploration of the electrochemical detection performance of Co_3_O_4_ on HMIs.

### Electrochemical testing

#### Optimization of conditions

To achieve optimal electrochemical detection performance of Co_3_O_4_-X/GCE in the detection of HMIs, it is necessary to optimize the test conditions (e.g., buffer, pH, deposition time, and deposition voltage) prior to electrochemical detection. As shown in Fig. S1a, comparing the peak response current changes of two buffers (0.1 M PBS and 0.1 M NaAc-HAc) for Pb^2+^ and Cu^2+^, the peak currents of Pb^2+^ and Cu^2+^ are observed to reach their maximum values in 0.1 M NaAc-HAc. The strongest peak currents are obtained. Therefore, NaAc-HAc buffer is selected as the optimal electrolyte for this experiment. The pH of the electrolyte has an effect on the electrochemical tests. As shown in Fig. S1b, the peak response currents of Pb^2+^ and Cu^2+^ increase when the pH increases from 3.6 to 5.0 and reaches a maximum at pH = 5.0. The peak response currents of Pb^2+^ and Cu^2+^ decrease when the pH continues to increase to 5.6. This is due to the fact that when the pH is low, protonation occurs on the surface of the modified electrode material, affecting the adsorption capacity of the material for HMIs. When the pH is high, hydrolysis of HMIs occurs, producing hydroxide precipitates and thus affects the electrochemical detection signal. Therefore, 0.1 M NaAc-HAc buffer at pH = 5.0 is selected as the optimal electrolyte. The deposition time also has a significant effect on the electrochemical detection performance. As shown in Fig. S1c, when the deposition time is increased from 60 to 150 s, the peak response currents of Pb^2+^ and Cu^2+^ increase consequently, reaching a maximum at a deposition time of 150 s. When the deposition time is continued to be increased to 180 s, there is no significant increase in the peak currents indicate that the active adsorption sites on the surface of the modified electrode material has reached the saturated. Therefore, 150 s is selected as the optimal deposition time. The deposition voltage is also one of the key factors affecting the electrochemical test, and the deposition voltage test was carried out at a deposition time of 150 s. As shown in Fig. S1d, when the deposition voltage decreases from -0.8 V to -1.0 V, the peak response currents of Pb^2+^and Cu^2+^continue to increase, and reach the maximum value when the deposition voltage is -1.0 V. As the potential continues to decrease to -1.2 V, the peak currents also decrease gradually. This is due to the fact that at the more negative potentials, Pb^2+^and Cu^2+^are reduced simultaneously with the reduction of H_2_O in the electrolyte solution to produce H_2_. This is then attached to the surface of the electrode and will hinder the deposition of HMIs on the electrode surface, thereby causing a decrease in the value of the peak current of dissolution. Therefore, -1.0 V is selected as the optimal deposition potential. From the optimal test conditions obtained from the above analysis, we perform the following electrochemical tests.

#### Electrochemical detection of Pb^2+^ and Cu^2+^

**Fig. 4:**
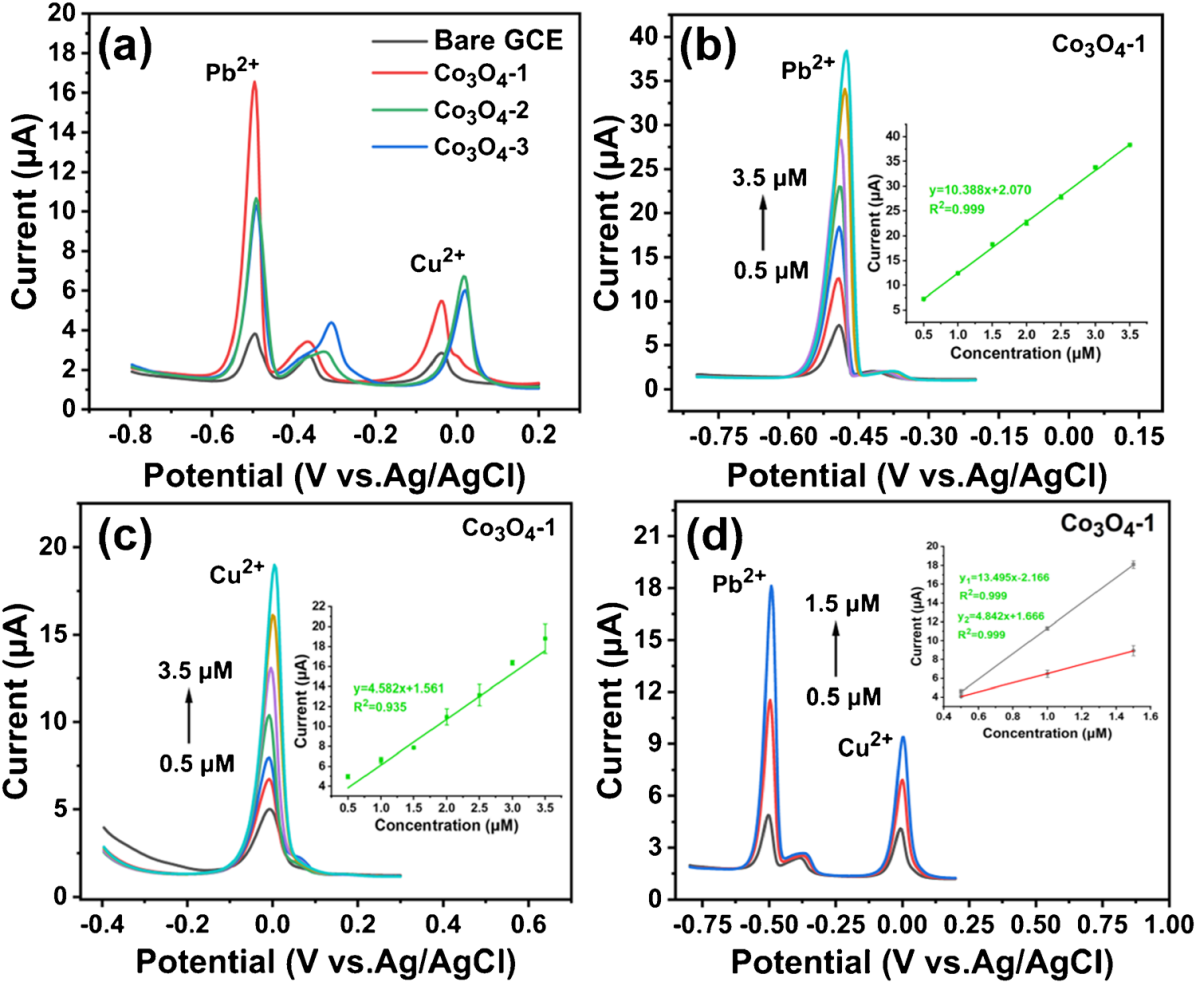
DPASV plots of (**a**) 1.0 μM Pb^2+^ and Cu^2+^, (**b-c**) individual detection and (**d**) simultaneous detection of different concentrations of Pb^2+^ and Cu^2+^ by bare electrode and Co_3_O_4_-X/GCE in 0.1 M pH = 5 NaAc-HAc. Inset: the corresponding calibration curves for the detection of Pb^2+^ and Cu^2+^, respectively. The error bars represent the standard errors of the three measurements

To deeply investigate the electrochemical performance of Co_3_O_4_-X materials for the detection of HMIs, a series of modified electrode active materials (Co_3_O_4_-X/GCE) are prepared for electrochemical testing, denoted as Co_3_O_4_-1/GCE, Co_3_O_4_-2/GCE and Co_3_O_4_-3/GCE, respectively. As shown in Fig. 4a, bare GCE and Co_3_O_4_-X/GCE are used to detect Pb^2+^ and Cu^2+^ under optimal test conditions. From the figure, the peak response currents of Co_3_O_4_- X/GCE are higher than that of bare GCE for both Pb^2+^ and Cu^2+^, and the peak response current of Co_3_O_4_-1/GCE is the largest. Weak peaks were observed in the range of -0.4 V to -0.3 V due to the interaction of heavy metal ions, probably caused by the formation of Pb-Cu intermetallic compounds during the deposition process [48]. Therefore, we select Co_3_O_4_-1/GCE as the modified electrode active material for the following electrochemical tests.

Figure 4b-c demonstrate the DPASV peak response currents of Co_3_O_4_-1/GCE for the detection of Pb^2+^ and Cu^2+^, respectively. The linear response of the peak currents to the concentration is observed in the range of 0.5–3.5 μM for both, with dissolution peak potentials of Pb^2+^ and Cu^2+^ estimated to be approximately -0.492 V and -0.008 V, respectively. From the figure, the peak response currents of Pb^2+^ and Cu^2+^ increase with the increase of concentration, and the current response signals of Pb^2+^ and Cu^2+^ show a good linear relationship with the concentration. The corresponding linear regression are I_pa_(μA) = 10.388C_Pb_^2+^(μM) + 2.070 (R^2^ = 0.999) and I_pa_(μA) = 4.582C_Cu_^2+^(μM) + 1.561 (R^2^ = 0.935), with the limits of detection (LOD, S/N = 3) of 2.38 nM and 31.08 nM, respectively, and limits of quantification (LOQ S/N = 10) are 7.94 nM and 103.60 nM, respectively (Table S1). In addition, a distance of about 0.5 V between the dissolution peak potentials of Pb^2+^ and Cu^2+^. Therefore, Pb^2+^ and Cu^2+^ can theoretically be detected simultaneously.

Figure 4d shows the DPASV peak response current of Co_3_O_4_-1/GCE for the simultaneous detection of Pb^2+^ and Cu^2+^. The results demonstrate a linear relationship between the peak response current and the concentration, with a range of 0.5–1.5 μM. Additionally, the dissolution peak potentials of Pb^2+^ and Cu^2+^ are about -0.504 V and -0.008 V, respectively. Co-deposition of HMIs on the active sites affects the electrochemical detection signal when Co_3_O_4_-1/GCE is used for simultaneous detection of Pb^2+^ and Cu^2+^ in solution as compared to detection alone. Interaction effects between different metals, such as competition for deposition and formation of intermetallic compounds, and these interaction effects may affect the determination of HMIs during the stripping process, and the HMIs compete with each other for the active sites on the electrode surface. However, due to the limited number of active sites, there will be saturation of the linear response range of HMIs, and thus a narrower linear response range is observed [49]. When Co_3_O_4_-1/GCE simultaneously detects the concentrations of Pb^2+^ and Cu^2+^, it improves the sensitivity of Pb^2+^ and Cu^2+^ compared to separate detection. This may be due to the formation of a Pb film during the deposition process, thereby would increase the sensitivity to Cu^2+^. With further increase in Pb^2+^ the peak current of Cu^2+^ also increases. When the Pb^2+^ concentration reaches a certain value, there may be surface saturation in the formation of Pb film. As a result, the peak current of Cu^2+^ tends to stabilize, forming a good linear relationship [50]. From the figure, the peak response current of Co_3_O_4_-1/GCE for simultaneous detection of Pb^2+^ and Cu^2+^ increase with the increase of concentration, and the current response signals of Pb^2+^ and Cu^2+^ show an excellent linear relationship with the concentration. The corresponding linear regression equations are I_pa_(μA) = 13.495C_Pb_^2+^(μM)-2.166 (R^2^ = 0.999) and I_pa_(μA) = 4.842C_Cu_^2+^(μM) + 1.666 (R^2^ = 0.999), and the limits of detection (LOD, S/N = 3) are 9.77 nM and 14.97 nM for Pb^2+^ and Cu^2+^, respectively, and these values are significantly lower than the standard values provide by the World Health Organization(the maximum permissible concentration for Pb in potable water is 48.26 nM, and that for Cu is 31.47 nM). The limits of quantification (LOQ, S/N = 10) of 32.55 nM and 49.90 nM for Pb^2+^ and Cu^2+^, respectively. A comparison of the Co_3_O_4_-1/GCE electrochemical sensor constructed in this work with other previously reported modified electrode materials, as shown in Table 1, reveals that it exhibits excellent electrochemical detection performance. This provides a reference value for practical applications.

**Table 1:** Comparison of electrochemical detection performance of other modified electrode active materials for simultaneous detection of Pb^2+^ and Cu^2+^

Electrode	Method	HMIs	LOD (nM)	Ref
alk-Ti_3_C_2_/GCE	SWASV	Pb^2+^ Cu^2+^	41 32	[51]
rGO/SMOF/PEI/SPCE	DPASV	Pb^2+^ Cu^2+^	25 55	[52]
Co-TMC4R-BDC/GCE	SWASV	Pb^2+^ Cu^2+^	44 67	[53]
ZIF-8-CS/GCE	DPASV	Pb^2+^ Cu^2+^	61.9 108	[54]
Fe_3_O_4_@SiO_2_/GCE	DPASV	Pb^2+^ Cu^2+^	16.5 79.4	[55]
Co_3_O_4_-1/GCE	DPASV	Pb^2+^ Cu^2+^	9.77 14.97	**This** **work**

In summary, the electrochemical sensor constructed by Co_3_O_4_-1/GCE has excellent detection performance. Conductivity and adsorption during electrochemical reactions (deposition and dissolution) are essential for excellent performance in electrochemical detection. Therefore, the excellent electrochemical sensing performance of the Co_3_O_4_-1/GCE constructed may be due to the excellent electrical conductivity of the active material Co_3_O_4_-1, the excellent adsorption performance, or both. To elucidate the key factors affecting the excellent performance of Co_3_O_4_-1/GCE for electrochemical detection of HMIs, the conductivity and adsorption performance of Co_3_O_4_-X /GCE as a modified electrode are analyzed.

### Electrochemical characterization

**Fig. 5:**
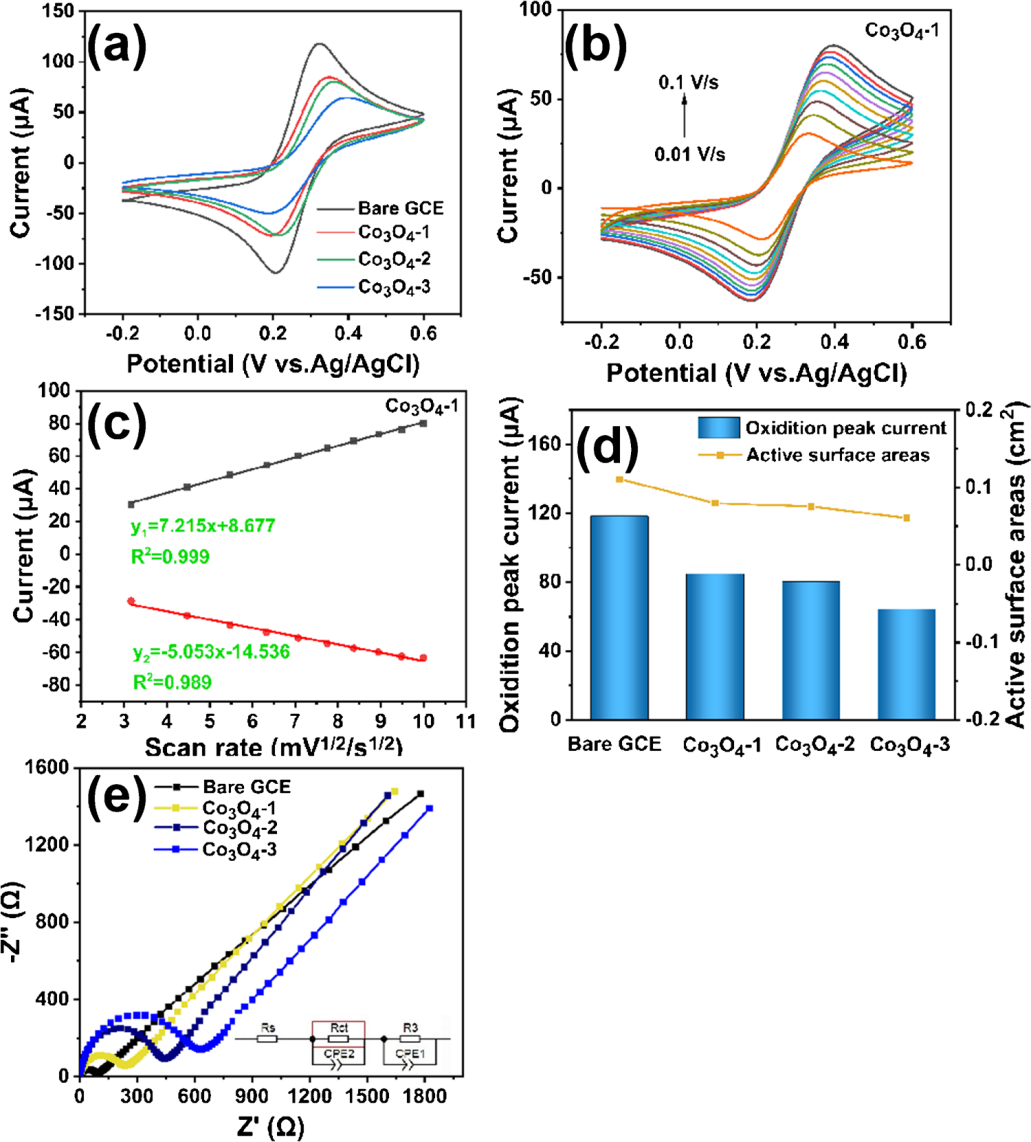
In a 5 mM [Fe(CN)_6_]^3−/4−^ solution containing 0.1 M KCl, (**a**) CV curves of bare electrode and Co_3_O_4_-X/GCE at a scan rate of 0.1 V/s, (**b**) CV curves of Co_3_O_4_-1/GCE at a scan rate range of 0.01–0.1 V/s, (**c**) peak redox Co_3_O_4_-1/GCE current with square root variation of scan rate, (**d**) histogram of peak redox current and active surface area. (**e**) EIS plots of bare electrode and Co_3_O_4_-X/GCE in 5 mM [Fe(CN)_6_]^3−/4−^ solution containing 0.1 M KCl in the frequency range 0.01–10^5^ Hz

To investigate the electrochemical activity of Co_3_O_4_-X materials and the factors influencing the performance of detecting HMIs, all samples as a series of modified electrodes. As shown in Fig. 5a, the CV curves of Co_3_O_4_-X/GCE in 5 mM [Fe(CN)_6_]^3−/4−^ solution containing 0.1 M KCl all show lower peak redox currents than bare GCE, indicating that the conductivity of Co_3_O_4_-X/GCE as a modified electrode is less than ideal. Since the above results show that the peak response currents of all modified electrodes to Pb^2+^ and Cu^2+^ are greater than those of the bare GCE (Fig. 4a). We hypothesize that the factors influencing the electrochemical activity and the performance of detecting HMIs of the Co_3_O_4_-X material are related to the adsorption properties. Compared with the other two modified electrodes, Co_3_O_4_ -1/GCE has the largest peak response current as a modified electrode, indicating that Co_3_O_4_-1/GCE has better electrochemical detection performance. Therefore, we select Co_3_O_4_-1/GCE as the modified electrode to investigate its kinetic behavior. Figure 5b shows the CV curves of Co_3_O_4_-1/GCE measured at 10–100 mV/s, and it can be clearly observed that the peak oxidation (reduction) current of Co_3_O_4_-1/GCE increases (decreases) with the increase of scan rate.

The magnitude of the peak oxidation (reduction) current at different scan rates is fitted to obtain the corresponding linear relationship between the square root of the scan rate (V^1/2^) and the peak cathodic (or anodic) current (I_pc_). As shown in Fig. 5c, the peak oxidation (reduction) current increases (decreases) with the scanning rate (R_O_^2^ = 0.999, R_R_^2^ = 0.989), indicating that the redox process on the Co_3_O_4_-1/GCE surface is under diffusion control. The Randles-Sevcik equation is used to calculate the effective electrochemically active surface area of the electrode surface. As shown in Fig. 5d, with the order of magnitude as Bare GCE (0.1107 cm^2^) > Co_3_O_4_-1/GCE (0.0795 cm^2^) > Co_3_O_4_-2/GCE (0.0753 cm^2^) > Co_3_O_4_-3/GCE (0.0604 cm^2^). In Co_3_O_4_-X/GCE, compared with the other two modified electrodes, the Co_3_O_4_-1/GCE electrode exhibits a greater electrochemically active surface area than the other two modified electrodes. This results in enhanced adsorption of HMIs and improved electrochemical detection performance.

To investigate the influence of the conductivity and charge transfer ability of bare GCE and Co_3_O_4_-X/GCE on the interfacial properties, EIS analysis on bare GCE and Co_3_O_4_-X/GCE. As shown in Fig. 5e, the Nyquist plots of bare GCE and Co_3_O_4_-X /GCE are fitted with the Randle equivalent circuit. The semicircle diameters of the fitted curves can represent the charge transfer resistance (R_ct_) magnitude in the order of Bare GCE (81.37 Ω) < Co_3_O_4_-1/GCE (250.00 Ω) < Co_3_O_4_-2/GCE (738.02 Ω) < Co_3_O_4_-3/GCE (1326.10 Ω). The results show that the R_ct_ of Co_3_O_4_-X/GCE is larger than that of both bare GCE, indicating that the conductivity of Co_3_O_4_-X/GCE is less than ideal. This finding is consistent with the previous CV results. Among Co_3_O_4_-X/GCE, Co_3_O_4_-1/GCE has the smallest R_ct_, indicating that this modified electrode has the fastest electron transfer rate.

### Adsorption capacity test

Based on the above CV curves and EIS analysis, the excellent electrochemical performance of Co_3_O_4_-1/GCE for the detection of Pb^2+^ and Cu^2+^ may be attributed to the best adsorption performance of Co_3_O_4_-1/GCE. A series of electrochemical ion adsorption experiments are performed to test this inference.

**Fig. 6:**
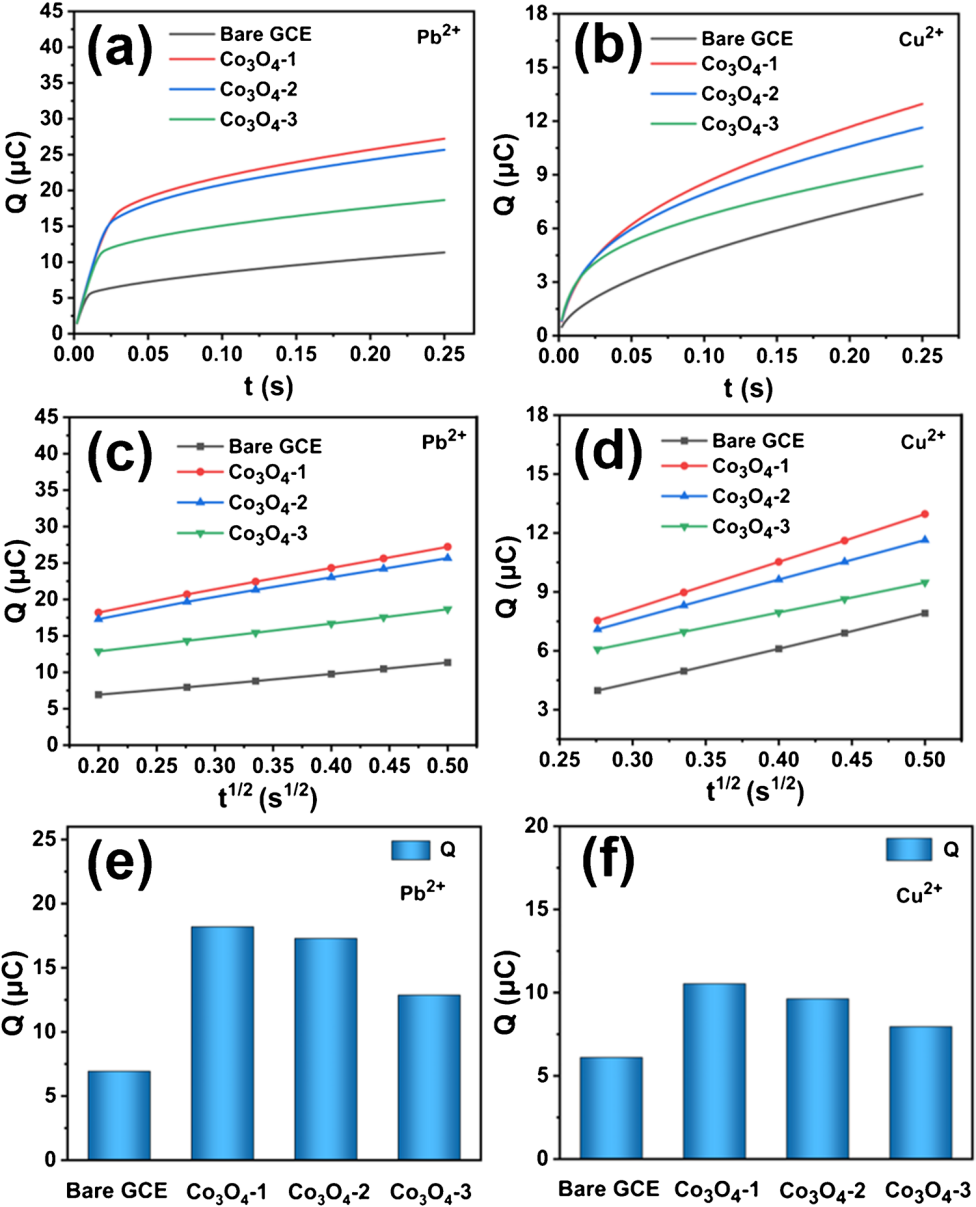
Plots of (**a-b**) timing curves, (**c-d**) variation of adsorption capacity with square root of adsorption time, and (**e–f**) histograms of adsorption Q magnitude for bare electrode and Co_3_O_4_-X/GCE on 2.0 μM

Figure 6a-b shows the adsorption profiles of Bare GCE and Co_3_O_4_-X/GCE for the detection of 2 μM Pb^2+^ and Cu^2+^ in 0.1 M NaAc-HAc, respectively. The square root variation of their adsorption capacity with adsorption time is shown in Fig. 6c-d. From the figure, the adsorption capacity of both the bare electrode and Co_3_O_4_-X/GCE as modified electrode increase with the increase of adsorption time, and the adsorption capacity of all modified electrodes is higher than that of the bare electrode. As shown in Fig. 6e-f, the adsorption amounts are in the order of Co_3_O_4_ -1/GCE (Q_Pb_^2+^ = 18.19 μC, Q_Cu_^2+^ = 7.54 μC) > Co_3_O_4_-2/GCE (Q_Pb2+_ = 17.29 μC, Q_Cu_^2+^ = 7.09 μC) > Co_3_O_4_-3/GCE (Q_Pb_^2+^ = 12.87 μC, Q_Cu_^2+^ = 6.07 μC) > Bare GCE (Q_Pb_^2+^ = 6.92 μC, Q_Cu_^2+^ = 3.98 μC). Among Co_3_O_4_-X/GCE, Co_3_O_4_-1/GCE show the highest adsorption as a modified electrode (Table S2), indicating that Co_3_O_4_-1/GCE has the strongest adsorption capacity for Pb^2+^ and Cu^2+^.Therefore, the distinctive Co_3_O_4_-1 microsphere structure composed of stacked fiber increases the active adsorption sites of the material, facilitating the adsorption of HMIs. The existence of O_V_ would also enhance the electrochemical detection performance of HMIs.

In summary, the excellent electrochemical detection performance of this electrode is mainly attributed to:(1) Compared to the other two materials, the distinctive Co_3_O_4_-1 microsphere structure composed of stacked fibers will enhance the active adsorption sites of the material, thereby facilitating the adsorption of HMIs. (2) The better electrical conductivity and larger electrochemically active surface area of Co_3_O_4_-1/GCE will accelerate the solution ion and charge transport in the solution, realizing efficient detection of Pb^2+^ and Cu^2+^. (3) The presence of O_V_ also promotes the adsorption of ions, thereby realizing efficient detection.

### Sensing mechanism research

The DPASV analytical method is utilized for the detection of Pb^2+^ and Cu^2+^ in the system, and the main processes involved in the assay include pre-enrichment and stripping. In this work, Co^2+^ and Co^3+^ in Co_3_O_4_-1 may be involved in the redox process. The unique structure of Co_3_O_4_-1 microspheres consisting of stacked fibers will increase the active adsorption sites of the material, and this facilitates the pre-enrichment process of HMIs. The sensing mechanism process of Co_3_O_4_-1/GCE consists of three main portions. **Route 1**: The adsorbed Pb^2+^ and Cu^2+^ get electrons on the surface of the modified electrode and are reduced to Pb^0^ and Cu^0^. **Route 2**: The pre-enriched Pb^0^ and Cu^0^ are oxidized again by the reverse voltage to Pb^2+^ and Cu^2+^, and returned to the solution. At the same time, the electrons return to the electrode surface and thus the stripping peak current signal is detected. **Route 3**: The redox cycle between Co^2+^/Co^3+^ in the system can act as a bridge between the electrode and Pb^2+^ and Cu^2+^ [49], and promote the redox reaction between Pb^2+^ and Cu^2+^. Therefore, the detection performance of Co_3_O_4_-1/GCE for Pb^2+^ and Cu^2+^ can be enhanced.

### Interference, stability and repeatability studies

According to the above electrochemical test results, Co_3_O_4_-1/GCE has good electrochemical detection performance for Pb^2+^ and Cu^2+^.To further validate the electrochemical sensing performance of this material, the interference, stability and repeatability of Co_3_O_4_-1/GCE are investigated.

**Fig. 7:**
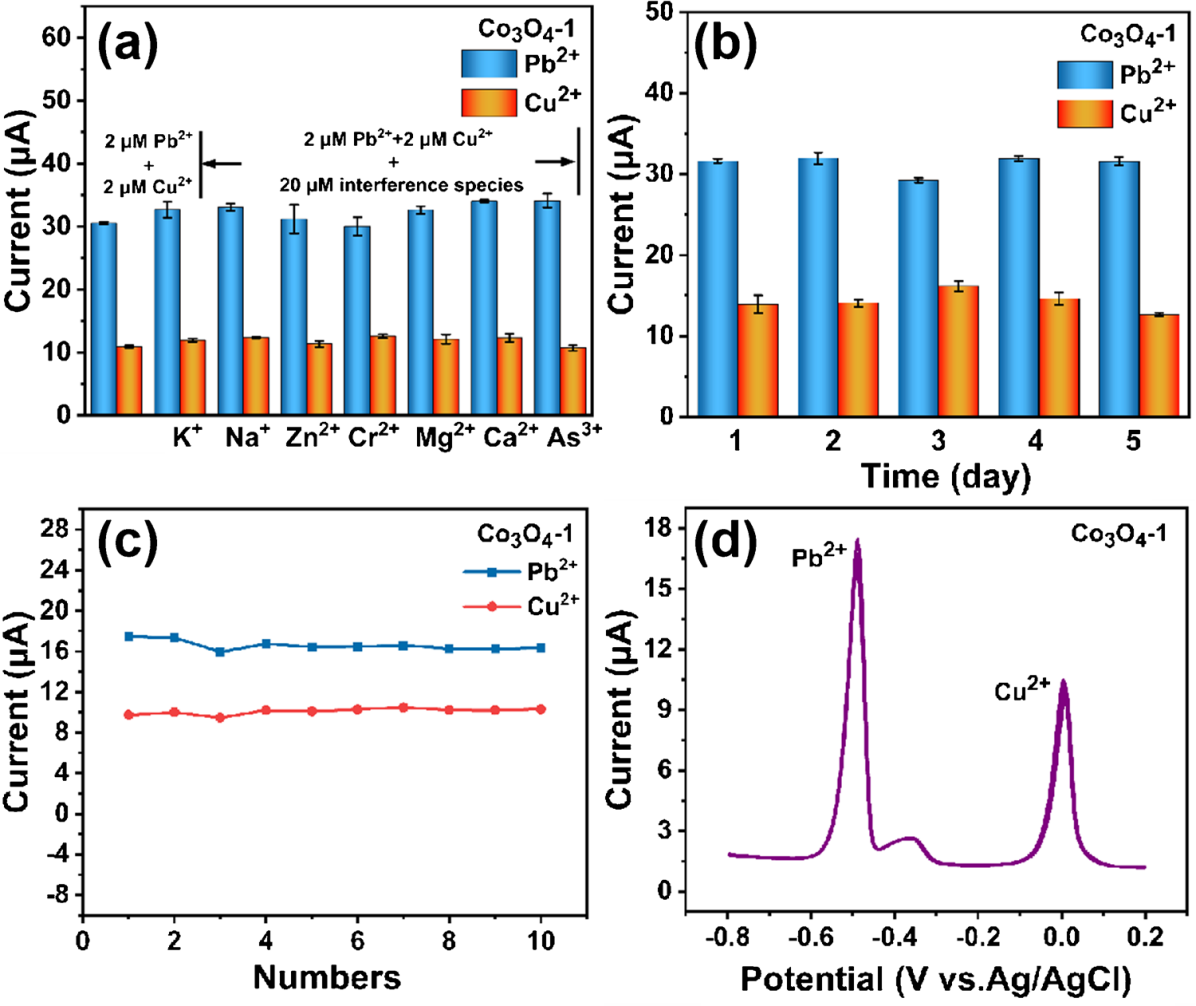
(**a**) Interference test, (**b**) Stability test: every other day, (**c-d**) Repeatability study: ten consecutive tests

As shown in Fig. 7a, the effect of Co_3_O_4_-1/GCE on the electrochemical detection of Pb^2+^ and Cu^2+^ are investigated by selecting K^+^, Na^+^, Zn^2+^, Cr^2+^, Mg^2+^, Ca^2+^ and As^3+^ as interfering ions, respectively. As shown in the figure, the peak response currents of Co_3_O_4_ -1/GCE to 2.0 μM Pb^2+^ and Cu^2+^ do not change significantly in the presence of 20 μM K^+^, Na^+^, Zn^2+^, Cr^2+^, Mg^2+^, Ca^2+^ and As^3+^. The high concentration of interfering ions does not affect the electrochemical testing of Co_3_O_4_-1/GCE for Pb^2+^ and Cu^2+^, indicating that Co_3_O_4_ -1/GCE has excellent anti-interference ability. The Co_3_O_4_-1/GCE is subjected to a five-day stability test, with measurements taken every other day. As shown in Fig. 7b, the peak current response of Co_3_O_4_-1/GCE to 2.0 μM Pb^2+^ and Cu^2+^ exhibit relatively little change. Figure 7c-d shows the Co_3_O_4_-1/GCE is detected continuously for ten times under the condition of 1.5 μM Pb^2+^ and Cu^2+^. As shown in the figure, the magnitude of the dissolved peak currents of Co_3_O_4_-1/GCE for Pb^2+^ and Cu^2+^ appear to be relatively stable after ten times of detection. This suggests that the Co_3_O_4_-1/GCE exhibits excellent stability, with RSD_Pb_^2+^ = 2.92%, RSD_Cu_^2+^ = 2.95%. Therefore, Co_3_O_4_-1/GCE is a promising candidate for the detection of HMIs in aqueous environments.

### Actual water sample tests

To ascertain the practical applicability of the constructed Co_3_O_4_-1/GCE electrochemical sensor, Co_3_O_4_-1/GCE was employed as a modified electrode to detect Pb^2+^ and Cu^2+^ in real water samples. As shown in Table S3, Three actual water samples were selected for analysis, including samples from our laboratory water supply, Lake 1, and Lake 2. The collected water samples are each diluted with 0.1 M NaAc-HAc solution (pH = 5.0) in a ratio of 1:9 by volume.

Calculations reveal that after the addition of 1.0 μM Pb^2+^ and Cu^2+^ to each of the three water samples, the peak response currents corresponding to their ions do not differ much, and the recoveries of Pb^2+^ and Cu^2+^ range from 91.7%-100.2% and 91.2%-115.2%, respectively (Table S3). The results show that Co_3_O_4_-1/GCE is reliable for detecting HMIs in real water samples. Therefore, the Co_3_O_4_-1/GCE electrochemical sensor has excellent reference value for practical applications.

## Summary

In this study, a series of MOF-derived Co_3_O_4_ materials is successfully synthesized by a surfactant micelle template solvothermal method. The electrochemical detection performance of Pb^2+^ and Cu^2+^ as modified electrode active materials in aqueous environments is then investigated. By adjusting different mass ratios of alkaline modifiers, the morphological microstructures of Co_3_O_4_-X exhibit a transition from distinctive microspheres composed of stacked fibers to rods. Co_3_O_4_-1/GCE has more excellent electrical conductivity and large electrochemically active surface area compared to the other two materials. It will accelerate the ion and charge transport in solution, thereby favoring the adsorption of HMIs and improving the electrochemical detection performance. Furthermore, Co_3_O_4_-1/GCE shows the largest peak currents in response to Pb^2+^ and Cu^2+^. This could be attributed to the distinctive microsphere structure composed of stacked fibers that enhances the active adsorption sites of the material and facilitates the adsorption of HMIs. The presence of O_V_ also promotes the adsorption of ions for efficient detection. The linear response range of Co_3_O_4_-1/GCE for the simultaneous detection of Pb^2+^ and Cu^2+^ is 0.5–1.5 μM, and the limits of detection (LOD, S/N = 3) are 9.77 nM and 14.97 nM, respectively. Co_3_O_4_-1/GCE also has excellent anti-interference ability, stability and reproducibility. In this work, the design of Co_3_O_4_-1/GCE electrochemical sensors can be used to detect HMIs in aqueous environments for practical applications.

## Supplementary information

Below is the link to the electronic supplementary material.

Supplementary file1 (DOCX 686 KB)

## Data Availability

All data in this study are included in the main paper and its Supplementary Information files.
