# Supplementary material for: Development of MOF-derived Co3O4 microspheres composed of fiber stacks for simultaneous electrochemical detection of Pb2+ and Cu2+
Source: Mikrochim Acta. 2024 Aug 17;191(9):542. doi: 10.1007/s00604-024-06623-7 (PMC11330412; doi:10.1007/s00604-024-06623-7)
Supplement: Supplementary file 1 — Supplementary file1 (DOCX 686 KB) [file 604_2024_6623_MOESM1_ESM.docx]

**Supplementary Materials for**

# Development of MOF-derived Co_3_O_4_ microspheres composed of fiber stacks for simultaneous electrochemical detection of Pb^2+^ and Cu^2+^

Jieli Guo^a^, Jin Li^a^, Xiujing Xing^b^, Wei Xiong^a,^*^^[[1]](#footnote-0)^*^*, Hao Li^c,*^

*^a^ Key Laboratory of Novel Biomass-Based Environmental and Energy Materials in Petroleum and Chemical Industry, Hubei Key Laboratory of Novel Reactor &Green Chemical Technology, School of Chemistry and Environmental Engineering, Wuhan Institute of Technology, Wuhan 430205, China*

*^b^ Chemistry Department, University of California, Davis 95616 United States*

*^c^ Advanced Institute for Materials Research (WPI-AIMR), Tohoku University, Sendai 980-8577, Japan*


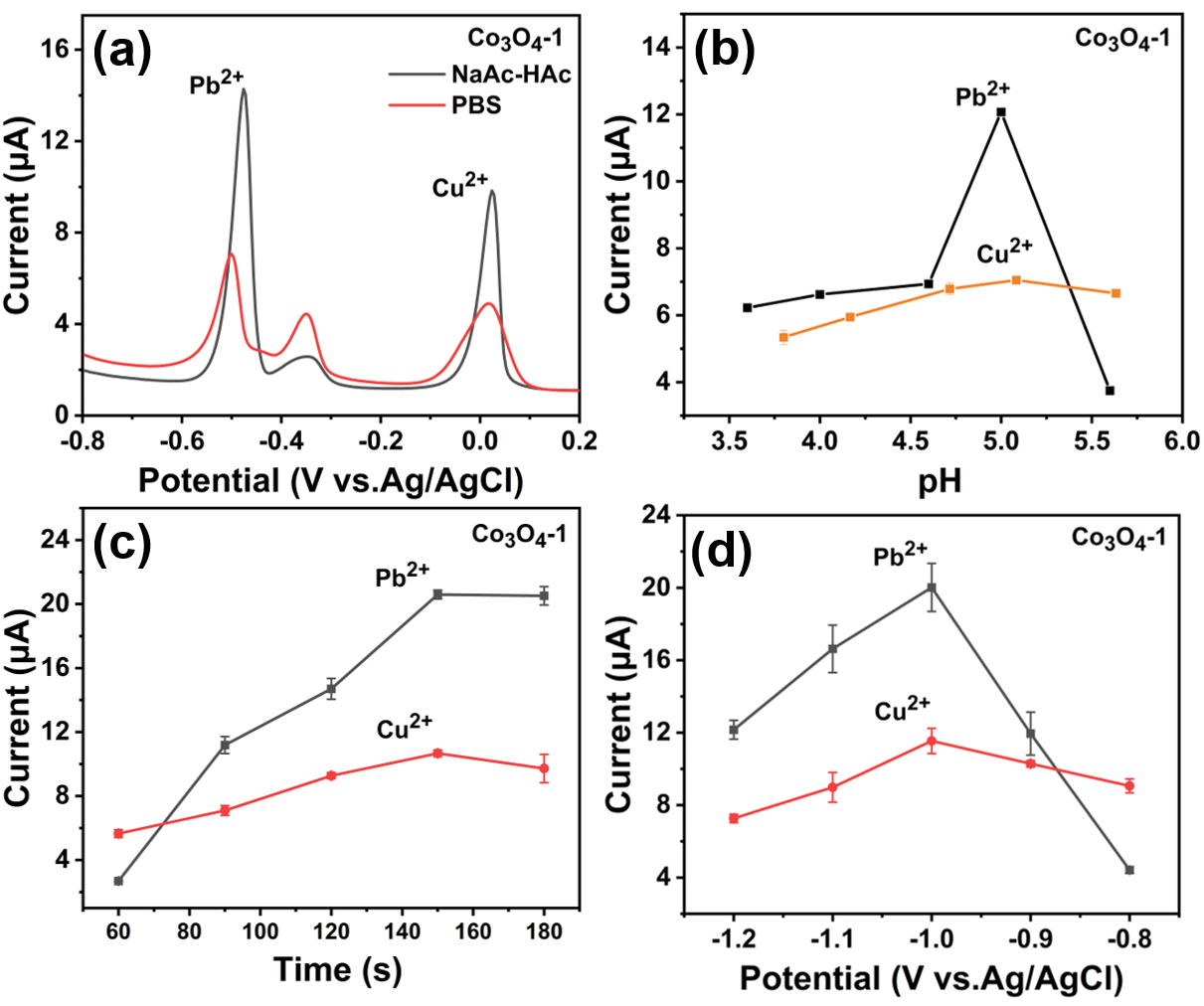


**Fig. S1** Optimized test conditions:**(a)** electrolyte, **(b)** pH, **(c)** deposition time, and **(d)** deposition voltage. The effects on the Co_3_O_4_-1/GCE current signals are investigated separately. The error bars represent the errors of the three measurements.


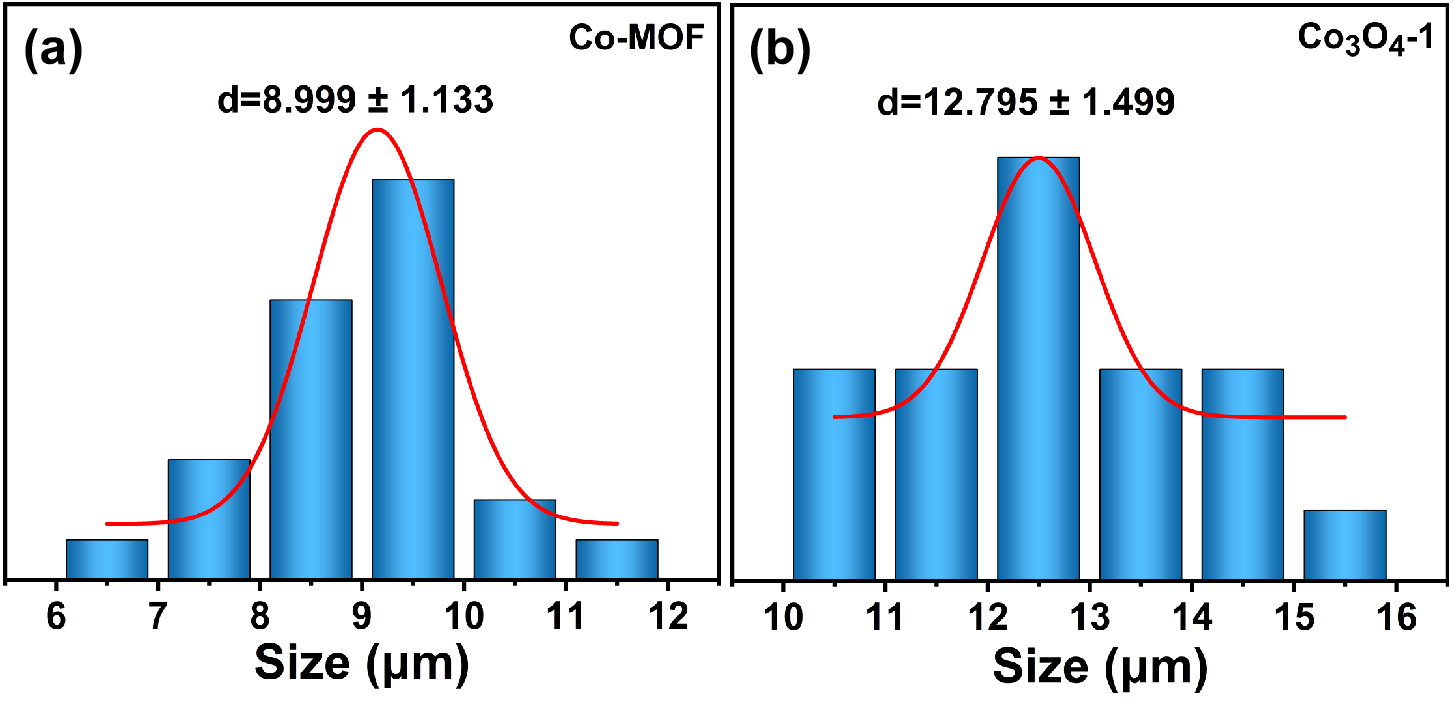


**Fig. S2 (a)** Particle size distribution of Co-MOF, **(b)** Particle size distribution of Co_3_O_4_-1.

**Table S1** LOD and LOQ values of Co_3_O_4_-1/GCE for Pb^2+^ and Cu^2+^ individually/simultaneously under the same test conditions

| analyte | LOD (nM) | LOQ (nM) |
| --- | --- | --- |
| individual testing Pb^2+^ | 2.38 | 7.94 |
| Cu^2+^ | 31.08 | 103.60 |
| simultaneous testing Pb^2+^ | 9.77 | 32.55 |
| Cu^2+^ | 14.97 | 49.90 |

**Table S2** Comparison of adsorption magnitude of bare electrode and Co_3_O_4_-X/GCE

| Working electrode | Q_Pb_^2+^ | Q_Cu_^2+^ |
| --- | --- | --- |
| Bare GCE | 6.924 | 3.978 |
| Co_3_O_4_ -1/GCE | 18.19 | 7.539 |
| Co_3_O_4_ -2/GCE | 17.29 | 7.093 |
| Co_3_O_4_ -3/GCE | 12.87 | 6.074 |

**Table S3** Detection recoveries of Co_3_O_4_-1/GCE for the detection of Pb^2+^ and Cu^2+^ in three water samples

| Real samples | Metal ions | Add (μM) | Found (μM) | Recovery (%) |
| --- | --- | --- | --- | --- |
| Sample 1 | Pb^2+^ | 1.0 | 0.917 | 91.7% |
|  | Cu^2+^ | 1.0 | 1.041 | 104.1% |
| Sample 1 | Pb^2+^ | 1.0 | 1.002 | 100.2% |
|  | Cu^2+^ | 1.0 | 0.912 | 91.2% |
| Sample 1 | Pb^2+^ | 1.0 | 0.956 | 95.6% |
|  | Cu^2+^ | 1.0 | 1.152 | 115.2% |

1. ^*^Corresponding author

   E-mail address: [xiongwei@wit.edu.cn](mailto:xiongwei@wit.edu.cn) (W. X.); [li.hao.b8@tohoku.ac.jp](mailto:li.hao.b8@tohoku.ac.jp) (H. L.)

   Tel/Fax: +86-27-87195001; +81-080-9363-8256​ [↑](#footnote-ref-0)
